# Synergistic efficacy of colistin and silver nanoparticles impregnated human amniotic membrane in a burn wound infected rat model

**DOI:** 10.1038/s41598-022-10314-9

**Published:** 2022-04-19

**Authors:** Nadia Wali, Aroosh Shabbir, Nadia Wajid, Nasir Abbas, Syed Zeeshan Haider Naqvi

**Affiliations:** 1 Institute of Molecular Biology and Biotechnology (IMBB), The University of Lahore, Defense Road Campus, Lahore, Pakistan; 2 Department of Pathology, Akhtar Saeed Medical and Dental College, Lahore, Pakistan; 3 Department of Statistics, Goverment Graduate College, Jhang, Pakistan

**Keywords:** Microbiology, Nanoscience and technology

## Abstract

Antimicrobials used to treat burn wound infections have become multidrug-resistant, thus delaying wound healing. When combined with silver nanoparticles, antibiotics create a multifaceted antibacterial mechanism of action to which bacteria are incapable of developing resistance. Similarly, the amniotic membrane has been found to lower the bacterial number. The purpose of the current study was to observe the antibacterial activity of combined topical colistin with silver nanoparticles and decellularized human amniotic membrane as a dressing in burn wounds infected with bacteria with the goal of promoting faster healing. Bacteria commonly isolated from burn wounds and the most sensitive topical antibiotic were identified. Colistin, silver nanoparticles and combined colistin with silver nanoparticles were impregnated into decellularized human amniotic membranes. These wound dressings were evaluated in third-degree multidrug-resistant bacterial infected thermal burns induced in rats. Out of a total of 708 pus samples from burn wounds, *Pseudomonas aeruginosa* was the most prevalent pathogen 308 (43.5%), followed by *Klebsiella pneumoniae* 300 (42.4%). Topical colistin was 100% sensitive for both bacteria. Overall, maximum wound contraction (*p* < 0.05), and increased collagen deposition (+++) with no isolation of bacteria from wound swabs were noted on day 21 for the combined colistin with silver nanoparticle-loaded human amniotic membrane dressing group. Our study concluded that the increased antimicrobial activity of the novel combination of colistin and silver nanoparticle-loaded decellularized human amniotic membrane manifested its potential as an effective burn wound dressing.

## Introduction

Infection is the main reason for mortality after severe burns despite developments in burn care management. Due to the lack of an epidermal barrier, bacteria can colonize and rapidly multiply after burn injuries^1,2^. *Pseudomonas aeruginosa* (*P. aeruginosa*), *Klebsiella pneumoniae* (*K. pneumoniae*) and *Staphylococcus aureus* (*S. aureus*) are among the commonly isolated pathogenic bacteria from infected burn victims. However, clinicians are faced with an additional arduous task of managing these antibiotic-resistant pathogenic infections^3^. Colistin has resurfaced as a last resort antibiotic for gram-negative bacterial infections resistant to other antibiotics^4^. It operates by binding to anionic lipid A molecules of gram-negative bacteria and alters the permeability of the cell wall, ultimately leading to cell leakage and death of bacteria^5^. However, the nephrotoxicity and neurotoxicity of colistin as a side effect are of serious concern. Similarly, antibiotic therapeutic choices have been severely constrained due to the steady rise in polymyxin resistant species^6,7^.

To make antibiotics that are already in use more effective, antibiotics have been combined with silver nanoparticles (AgNPs) to maximize the antibacterial effect^8^. AgNPs have adequate antibacterial action, making them excellent candidates for exploration as alternative agents to treat multidrug-resistant (MDR) infections^9^. Their particle size in nanometers is observed to have an increased surface area which can destroy the membrane and produce intracellular damage^10^. AgNPs vary from antibiotics as they attach to negatively charged proteins, RNA and DNA while posing minimal risk of bacterial resistance^11^.

In the last few decades, the human amniotic membrane (hAM) has received considerable attention due to its wide utilization in tissue regeneration and wound healing applications. It serves as an excellent biological dressing because it is biodegradable and promotes re-epithelization with antimicrobial and anti-inflammatory effects. It can be easily seeded with other cells for their growth due to its porous structure. The decellularization of the hAM removes all cellular components, while its basement membrane and extracellular matrix proteins remain unharmed. The decellularization process has the additional benefit of lowering the immunogenicity response with improved and consistent cellular proliferation and differentiation properties^12–14^.

Considering the unique abilities of AgNPs to combat MDR bacteria and useful biological dressing aspects of hAM, we hypothesized the combined antibacterial effects of colistin, AgNPs and decellularized human amniotic membrane (dHAM) in-vivo. Therefore, in the current study for the first time, we fabricated a novel burn wound dressing by using dHAM loaded with colistin and AgNPs. This study was conducted to evaluate the synergistic antibacterial activity of topical colistin, AgNPs and dHAM as a dressing in a burn wound-infected rat model that can expedite the wound healing process.

## Ethics statement

The department of Bioethics, Biosafety and Biosecurity of The University of Lahore, Lahore, approved the research and animal ethics (IMBB/UOL/21/001). The Helsinki declaration was followed for the acquisition and utilization of hAM for research, and animal handling and dissection were carried out according to ARRIVE guidelines. No placental tissues were procured from prisoners. Written informed consent was obtained from each patient enrolled for sampling. All methods were carried out in accordance with relevant named guidelines and regulations.

## Materials

Pus swabs (Microbiologics Inc., USA), and 6-well cell culture plates were purchased from SPL Life Sciences Co., Ltd., Korea. Liquid nitrogen was obtained from Media gas Pvt. Ltd., and de-ionized water from Ultra Pure, Pakistan. Colistin sulfate powder, formaldehyde, haematoxylin and eosin (H&E) staining were purchased from Merck (Sigma-Aldrich, Germany). Ketamine hydrochloride injection (50 mg, Ketasol) and xylazine injection (23.32 mg, Xylax) were procured from Indus Pharma Pvt. Ltd., and Mylab Pvt. Ltd., Pakistan respectively.

Blood agar, MacConkey agar, Mueller hinton agar (MHA) and all commercial antibiotic discs were purchased from Oxoid Ltd., Thermo Fischer Scientific, UK. Analytical profile index 20 E for biochemical tests and 0.5 McFarland standard were purchased from BioMérieux (France). The already prepared and characterized AgNPs (size 5–30 nm) from the *Aspergillus flavus* (*A. flavus*) source were procured from Dr Syed Zeeshan Haider Naqvi (Microbiology department, The University of Lahore, Lahore) for further study^15^. Hepatitis B virus (HBV), hepatitis C virus (HCV), human immunodeficiency virus (HIV) and syphilis screening kits performed by immunochromatography were procured from Standard Diagnostics Bioline, Abbott, Korea. The following machines were used in the experiment: VITEK 2 (BioMérieux, France) system, sonication (Sonic-Vibra cell, USA), lyophilizer (VaCo 2-Zirbus Technology, Germany), shaker (DLAB, China), Light microscope (Olympus, Japan) & Microtome (Leica, Germany).

## Methods

This experimental study was carried out at the Department of Institute of Molecular Biology and Biotechnology, The University of Lahore, Lahore, from December 2018 to January 2020. Only burn wound sample processing, identification of bacteria and phenotypic detection were performed at the Burn Care and Reconstructive Surgery Centre at Jinnah Hospital, Lahore.

Cesarean section, noncomplicated pregnancy, and patients with third-degree thermal burns were included in the study. However, features of neonatal infection, premature delivery or premature rupture of membranes, transmissible diseases (e.g., HBV, HCV, HIV & syphilis), other external sources of burns (chemical, electrical & radiation), and all degrees of burns other than third degree were excluded from the study.

### Identification and antibiotic sensitivity testing of commonly isolated bacteria from burn wound samples of patients

A total of 708 pure pus and pus swabs were collected from hospitalized burn ward patients, applied to Blood agar and MacConkey agar and incubated at 35  ±  2 °C for 16–18 h. Further standard microbiology protocols were used to identify the bacterial isolates. Antibiotic sensitivity testing was performed by the Kirby Bauer disc diffusion method by mixing one to two colonies of commonly isolated bacteria into a tube containing 5 mL of sterile normal saline (0.5 McFarland solution) and comparing it with 0.5 McFarland standard. Lawns of each bacterial suspension were made on MHA using sterile cotton swabs, and commercially available standard antimicrobial discs for gram-negative bacteria were applied on it. The discs employed were 10 µg of ampicillin, colistin, gentamicin, imipenem and meropenem; 5 µg of ciprofloxacin and levofloxacin; 20/10 µg of amoxicillin-clavulanate, cefepime, ceftazidime, aztreonam and amikacin; 10/10 µg of ampicillin-sulbactam; and 100/10 µg of piperacillin-tazobactam. The sensitivity plates were incubated at 35 ± 2 °C for 16–18 h, and zone sizes were interpreted according to Clinical and Laboratory Standards Institute (CLSI) 2020 criteria^16,17^.

Bacteria commonly isolated from burn wounds were considered for study. Three MDR bacteria (resistance to one agent in three or more antibiotic categories) from these commonly isolated bacteria with minimum inhibitory concentrations < 2 μg/mL (considered sensitive for colistin) by the VITEK 2 system were selected for further study^17,18^. Similarly, the maximum sensitivity exhibited by topical antibiotic against the commonly isolated bacteria was selected for further experimentation.

### Concentration determination of colistin and AgNPs in-vitro

The stock solutions of colistin and AgNPs were prepared by adding 150 mg of colistin sulfate powder and AgNP powder separately into 100 mL of de-ionized water (20 min of sonication of AgNPs suspension with vortexing repeatedly for 5 days) in screw-capped glass bottles. A volume of 15.6 mL from the respective stock solutions was picked up and then added individually into 50 mL of distilled water in other screw-capped bottles to make 0.468 mg/mL dilutions of colistin and AgNPs for final use^17,19,20^. The formula used to calculate dilutions of colistin and AgNPs can be found in Supplementary Fig. 1 online.

### Colistin and AgNPs impregnation into dHAM

All scheduled cesarean sections were included to take the placenta and were prescreened for transmissible diseases by screening kits. The amnion was isolated from the chorion, and the decellularization process was initiated by snap freezing, employing liquid nitrogen for 1 min to detach the monolayer of amnions' epithelium. Confirmation was performed through H&E staining of the samples and histological observation by light microscopy^21^.

A total of four (2 × 2 cm) lyophilized membranes were placed in 4 wells of a total 6-well (cell culture) plate. In the 1st well, 2 mL of distilled water was added as a negative control. Colistin (2 mL) was added to the 2nd well, the 3rd well with 2 mL of AgNPs and the 4^th^ well with 2 mL of AgNPs (1 mL) and colistin (1 mL), where in all of these cases, a colistin and AgNP dilution of 0.468 mg/mL was used. The plate was then kept overnight on the shaker.

### In-vivo efficacy of colistin and AgNPsimpregnated into dHAM in rats

Thirty Sprague Dawley rats were purchased from the animal unit of The University of Lahore, Lahore. The rats weighed approximately 200 g–250 g and were kept in a regulated room temperature (22 ± 2 °C) with unrestricted food and water for a week. Ketamine and xylazine were injected intraperitoneally to anesthetize these healthy rats. A solid iron bar of 2 × 2 cm was dipped in boiling water (at 100 °C) and thermal injury was induced by keeping this hot bar in contact for 30 s on the dorsum of the rat^22^. Evaluation of the wound was performed, and surgical debridement of the wound under general anesthesia was performed to remove necrosis the next day^23,24^.

Three MDR strains each for *P. aeruginosa* and *K. pneumoniae* (10^6^ CFU/mL) were inoculated through the intradermal route. After 24 h of contamination of rat wounds, the animals were randomly divided into five dressing groups: the positive control (without any treatment) group, negative control (distilled water-dHAM) group, colistin-dHAM group, AgNPs-dHAM group and colistin + AgNPs-dHAM group. All wounds were secured with bandage afterwards. The experiment was performed in triplicate.

The bandages were opened on days 7, 14 and 21, and wound contraction was noted. The wound area was retraced on millimeter scale graph paper from the tracing paper, and wound closure was expressed as a reduction in the percentage of original wound size.

% wound contraction on day X = [(area on day 0 – open area on day X)/area on day 0] × 100.

The American Veterinary Medical Association (AVMA) Guidelines for Euthanasia of Animals (2020) were followed. Tissue samples of these healed rat wounds were removed on day 21 and immediately fixed by dipping in 10% neutral buffered formalin, followed by routine histological processing. Tissues were subjected to microtomy with 4 μm cuts, and finally, H&E staining was performed. The histological analysis of wound healing was performed by a trained histopathologist. Re-epithelialization and other histological healing parameters were noted according to the following criteria:

1. Inflammatory response, featured by the presence of polymorphonuclear neutrophils
2. Granular tissue indicated by the presence of fibroblasts, myofibroblasts, and neovascularization
3. Fibrosis, indicated by the density of collagen fibers. A score was made for all parameters:

− = absent,

+ = mild presence,

++ = moderate presence,

and +++ = strong presence^25^.

### Determination of antimicrobial activity

The swabs were taken from all of the wounds on day 21 and were applied separately to the Blood and MacConkey media to observe the growth of any bacteria. Bacterial morphology, staining and biochemical testing were performed to identify the bacteria^26^.

### Statistical analysis

The analysis was carried out with the help of the Statistical Package for Social Sciences (SPSS) version 24 (IBM, USA). Percentages, frequencies, means, medians and standard deviations were calculated for variables wherever applicable. Data was observed to be normally distributed by applying Shapiro–Wilk statistical test. To compare the efficacy of five groups, one-way ANOVA was performed, and Dunnett's test was used for pairwise comparisons to observe the effect of time and treatment groupswith *p* < 0.05 was considered statistically significant.

## Results

### Frequency of bacteria isolated from burn wound samples of patients

Of the 708 total isolated bacteria from pure pus and pus swabs from burn wounds, *P. aeruginosa* was isolated a predominant isolate, 308 (43.5%), followed by *K. pneumoniae,* 300 (42.4%), as summarized in Table 1. However, there was minimal difference between their number of isolations and other gram-negative bacteria and gram-positive cocci were isolated less frequently.

**Table 1:** Frequency of bacteria isolated from burn wound patients (N = 708).

Bacteria	Number	%age	Proportion	SD
*P. aeruginosa*	308	43.5	0.44	0.02
*K. pneumoniae*	300	42.4	0.42	0.02
*P. mirabilis*	44	6.2	0.06	0.01
*S. aureus*	38	5.4	0.05	0.01
*A. baumannii*	18	2.5	0.03	0.01

*A. baumannii*, *Acinetobacter baumannii*; *P. mirabilis*, *Proteus mirabilis*; SD, standard deviation.

### Antimicrobial sensitivity testing against *P. aeruginosa & K. pneumoniae*

When topical antibiotics were considered, maximum susceptibility was exhibited by colistin 308 (100%) followed by gentamicin 141 (45.78%) for a total of 308 *P. aeruginosa* isolates. There were no isolates with intermediate susceptibility patterns in the case of *P. aeruginosa* (Table 2). Similarly, maximum susceptibility was presented by colistin 300 (100%) followed by gentamicin 97 (32.33%) for a total 300 *K. pneumoniae* isolates, with few isolates having intermediate susceptibility, as shown in Table 3.

**Table 2:** Antimicrobial sensitivity pattern against *P. aeruginosa* from burn wound patients (N = 308).

Antimicrobials	Sensitive				Intermediate	Resistant			
	Number	%age	Proportion	SD	Number	Number	%age	Proportion	SD
Colistin	308	100.00	1.00	0.00	0	0	0	0.00	0.00
Meropenem	154	50.00	0.50	0.03	0	154	50	0.50	0.03
Gentamicin	141	45.78	0.46	0.03	0	167	54	0.54	0.03
Amikacin	139	45.13	0.45	0.03	0	169	55	0.55	0.03
Cefoperazone-sulbactam	132	42.86	0.43	0.03	0	176	57	0.57	0.03
Imipenem	145	47.08	0.47	0.03	0	163	53	0.53	0.03
Piperacillin-tazobactam	132	42.86	0.43	0.03	0	176	57	0.57	0.03
Levofloxacin	72	23.38	0.23	0.02	0	236	77	0.77	0.02
Ciprofloxacin	86	27.92	0.28	0.03	0	222	72	0.72	0.03
Ampicillin-sulbactam	46	14.94	0.15	0.02	0	262	85	0.85	0.02
Cefepime	89	28.90	0.29	0.03	0	219	71	0.71	0.03
Amoxicillin-clavulanic acid	132	42.86	0.43	0.03	0	176	57	0.57	0.03
Ceftazidime	117	37.99	0.38	0.03	0	191	62	0.62	0.03

**Table 3:** Antimicrobial sensitivity pattern against *K. pneumoniae* from burn wound patients (N = 300).

Antimicrobials	Sensitive				Intermediate				Resistant					SD
	No	%age	Proportion	SD	Number		%age	proportion	SD		No	%age	proportion	
Colistin	300	100.00	1.00	0.00	0		0.00	0.00	0.00		0	0	0.00	0.00
Amikacin	137	45.67	0.46	0.03	6		2.00	0.02	0.01		157	52	0.52	0.03
Gentamicin	97	32.33	0.32	0.03	0		0.00	0.00	0.00		203	68	0.68	0.03
Cefoperazone-sulbactam	96	32.00	0.32	0.03	1		0.33	0.00	0.00		203	68	0.68	0.03
Meropenem	90	30.00	0.30	0.03	0		0.00	0.00	0.00		210	70	0.70	0.03
Imipenem	84	28.00	0.28	0.03	0		0.00	0.00	0.00		216	72	0.72	0.03
Piperacillin-tazobactam	72	24.00	0.24	0.02	0		0.00	0.00	0.00		228	76	0.76	0.02
Levofloxacin	54	18.00	0.18	0.02	0		0.00	0.00	0.00		246	82	0.82	0.02
Ciprofloxacin	53	17.67	0.18	0.02	1		0.33	0.00	0.00		246	82	0.82	0.02
Ampicillin-sulbactam	45	15.00	0.15	0.02	0		0.00	0.00	0.00		255	85	0.85	0.02
Cefepime	41	13.67	0.14	0.02	0		0.00	0.00	0.00		259	86	0.86	0.02
Aztreonam	25	8.33	0.08	0.02	0		0.00	0.00	0.00		275	92	0.92	0.02
Amoxicillin-clavulanic acid	12	4.00	0.04	0.01	0		0.00	0.00	0.00		288	96	0.96	0.01

### Wound healing of burn wounds in rats against *P. aeruginosa and K. pneumoniae*

**Figure 1:**
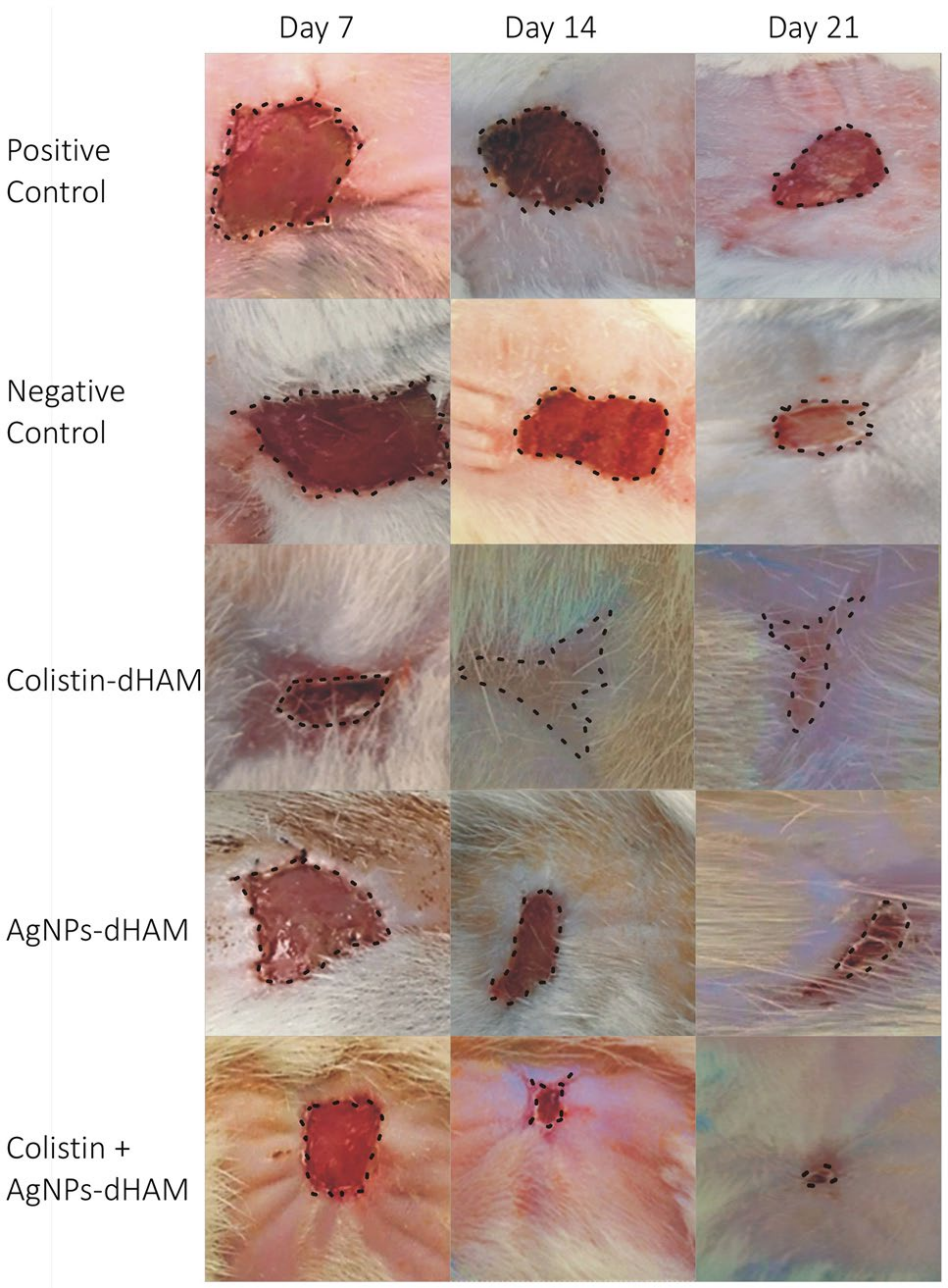
Wound healing in different dressing groups of rats against *P. aeruginosa* on days 7, 14 & 21.

**Figure 2:**
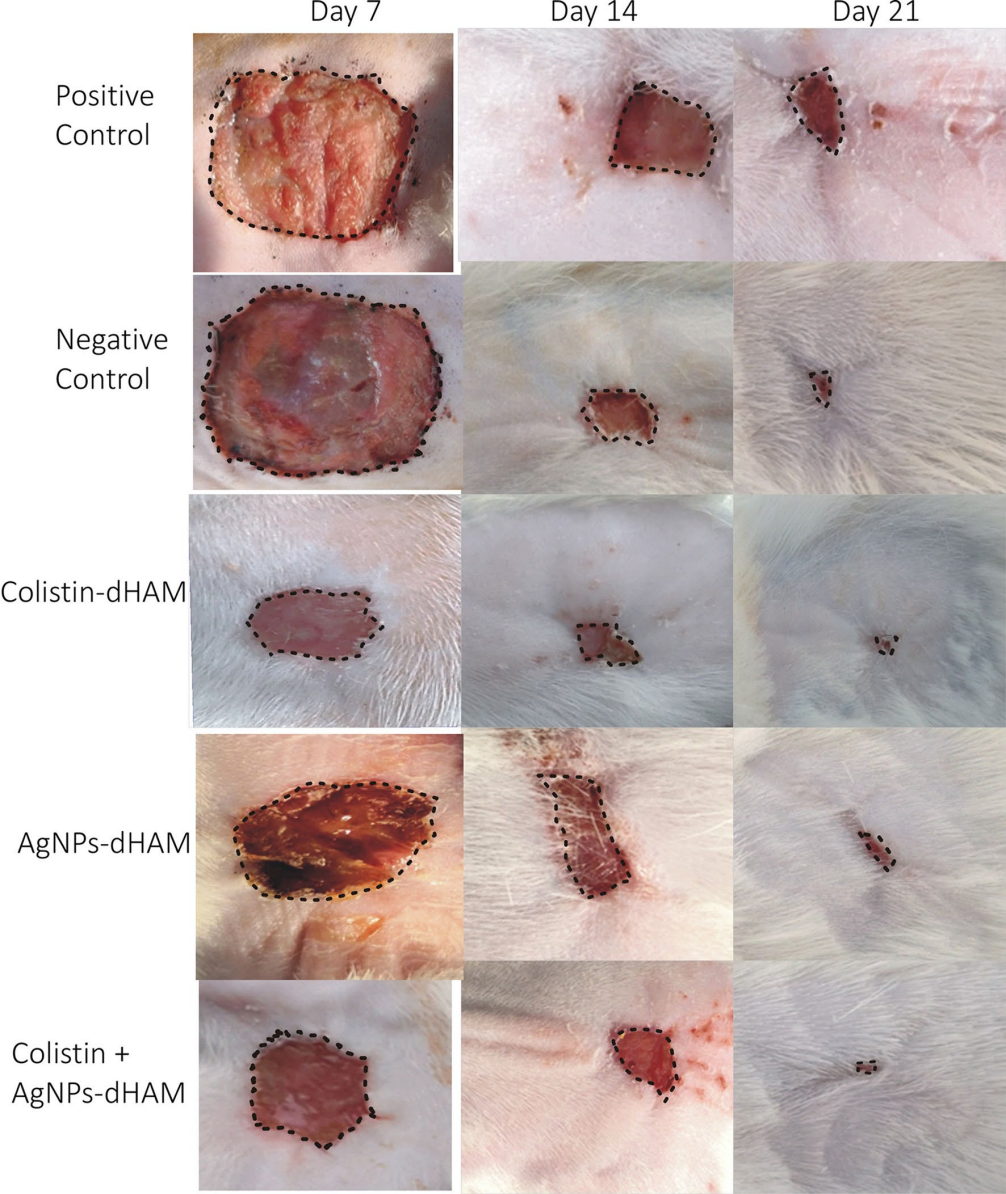
Wound healing in different dressing groups of rats against *K. pneumoniae* on days 7, 14 & 21.

The wound contractions of the different dressing groups used against *P. aeruginosa and K. pneumoniae* and the entire findings of the statistical analysis are reported in Table 4. The most significant changes were observed on day 7, where the colistin-dHAM dressing group initially accelerated wound contraction compared to the other dressing groups but finally achieved less contraction than the combined colistin + AgNPs-dHAM dressing group on day 21 for both bacteria. Furthermore, the different dressing groups under study were highly significant with regard to their performance (*p* < 0.05). The administration of combined colistin + AgNPs-dHAM dressing group in the burn wound rat model significantly (*p* < 0.05) increased the wound contraction rate compared to the positive control on day 21 for both *P. aeruginosa* and *K. pneumoniae*. Meanwhile, the contraction rate of the nontreated dressing group was only 41.3 ± 3.2% for *P. aeruginosa* & 64.7 ± 4.5% for *K. pneumoniae* on day 21 postburn. The efficacy of colistin-dHAM was insignificant (*p*= 0.108) versus synergistic efficacy of colistin + AgNPs-dHAM (*p* < 0.001) for *P. aeruginosa*. However, in the case of *K. pneumoniae*, colistin + AgNPs-dHAM was highly significant (*p* < 0.001) with respect to colistin-dHAM (*p* = 0.004). The wound contraction of various dressing groups is displayed in Figs. 1 and 2.

**Table 4:** Comparison of wound contraction in different treatment groups *against P. aeruginosa and K. pneumoniae.*

Days	Statistical measures	Treatment groups (zones of inhibition in mm)				
		Positive control (%age)	Negative Control (%age)	Colistin-dHAM (%age)	AgNPs-dHAM (%age)	Colistin + AgNPs-dHAM (%age)
***P. aeruginosa***						
^a^Comparison between and within groups (7^th^ day), *p* value = 0.000*						
7th day	Mean ± SD ^b^(*p* values)	10.0 ± 3.6	1.3 ± 0.6 (*p*: 0.295)	32.3 ± 11.0 (*p*: 0.014*)	18.3 ± 10.4 (*p*: 0.325)	25.0 ± 5.0 (*p*: 0.040*)
Comparison between and within groups (14th day), *p* value = 0.012*						
14th day	Mean ± SD (*p* values)	16.3 ± 3.2	21.0 ± 1.7 (*p*: 0.677)	36.0 ± 6.1 (*p*: 0.001*)	29.7 ± 1.5 (*p*: 0.039*)	33.0 ± 2.6 (*p*: 0.011*)
Comparison between and within groups (21st day), *p* value = 0.000*						
21st day	Mean ± SD (*p* values)	41.3 ± 3.2	52.7 ± 2.1 (*p*: 0.002*)	47.0 ± 2.6 (*p*: 0.108)	70.3 ± 1.5 (*p*: 0.000*)	87.7 ± 4.2 (*p*: 0.000*)
***K. pneumoniae***						
Comparison between and within groups (7^th^ day), *p* value = 0.000*						
7th day	Mean ± SD (*p* values)	1.0 ± 0.0	1.3 ± 0.6 (*p*: 0.996)	42.3 ± 2.5 (*p*: 0.000*)	32.3 ± 2.1* (*p*: 0.000*)	35.3 ± 0.6* (*p*: 0.000*)
Comparison between and within groups (14^th^ day), *p* value = 0.000*						
14th day	Mean ± SD (*p* values)	24.0 ± 1.0	38.0 ± 2.6 (*p*: 0.12*)	65.3 ± 2.5 (*p*: 0.000*)	52.0 ± 8.7 (*p*: 0.000*)	67.3 ± 3.8 (*p*: 0.000*)
Comparison between and within groups (21^st^ day), *p* value = 0.000*						
21st day	Mean ± SD (*p* values)	64.7 ± 4.5	73.0 ± 2.6 (*p*: 0.030*)	76.3 ± 1.5 (*p*: 0.004*)	82.0 ± 3.5 (*p*: 0.000*)	92.0 ± 3.0 (*p*: 0.000*)

All data are presented as mean ± SD. Superscript a. One-way ANOVA and b. Two-sided Dunnett’s test in which all other dressing groups are compared with the positive control. *p* < 0.05: statistically significant*

### Histological features of rat wound healing against *P. aeruginosa* and *K. pneumoniae*

**Figure 3:**
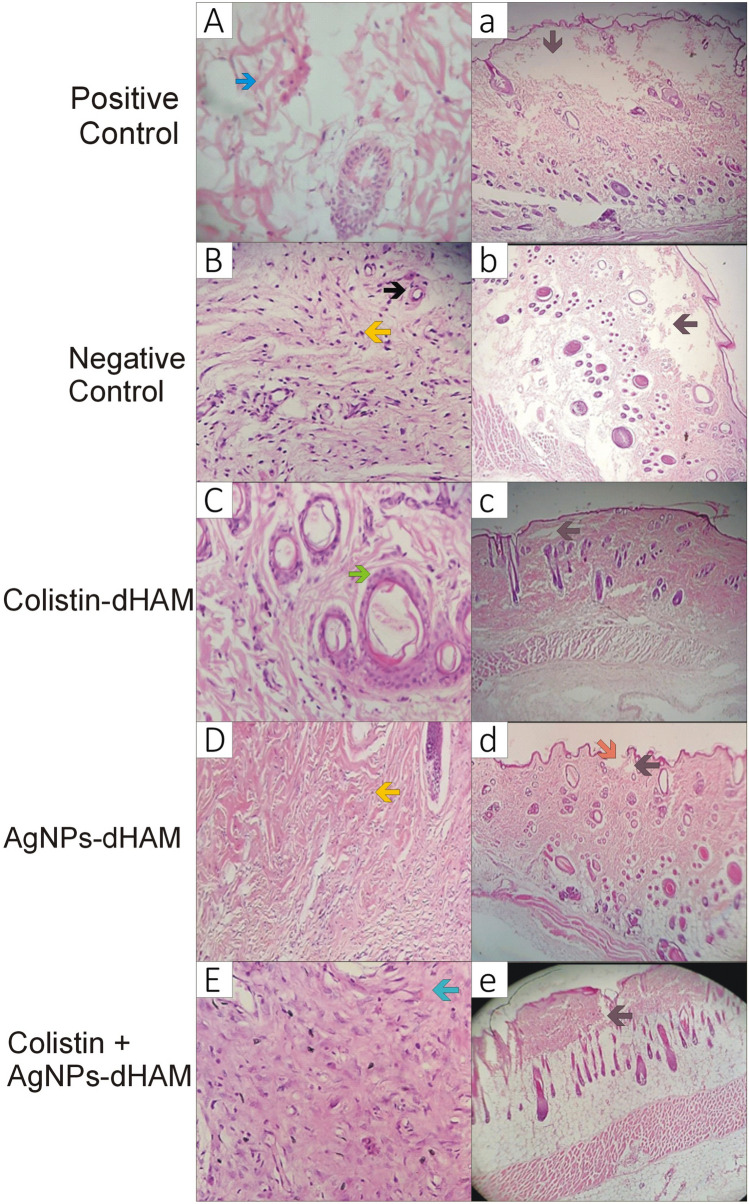
Microscopic evaluation of rat wound healing against *P. aeruginosa* on day 21. (**A**). positive control group with mild inflammation and mild disrupted collagen formation, (**B**). negative control group with strong inflammation and moderate collagen formation, (**C**). colistin-dHAM group with mild inflammation and collagen formation, (**D**). AgNPs-dHAM group with moderate inflammation and collagen formation with neovascularization, (**E**). colistin + AgNPs-dHAM group- moderate inflammation and neovascularization with dense collagen formation. Black arrow, neovascularization; blue arrow, collagen formation; yellow arrow, inflammatory cells; green arrow, hair follicles. Complete re-epithelialization in a, b, c & e except d (orange arrow). Dermal damage was visible (brown arrow) in all groups.

**Figure 4:**
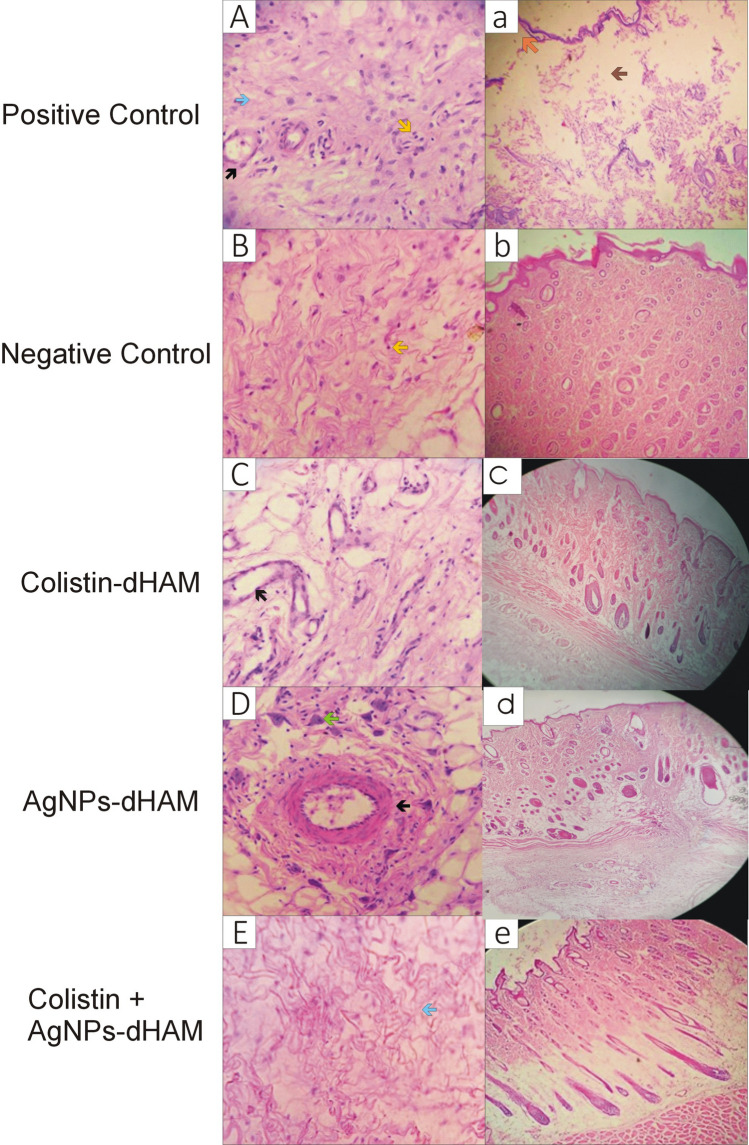
Microscopic evaluation of rat wound healing against *K. pneumoniae* on day 21*.* (**A**) positive control group with intense inflammatory infiltrate, mild neovascularization and moderate collagen formation, (**B**) negative control group with moderate inflammation and collagen formation, (**C**) colistin-dHAM group with moderate inflammation and strong presence of neovascularization, (**D**) AgNPs-dHAM group with mild presence of inflammation and numerous myofibroblasts, (**E**) colistin + AgNPs-dHAM group- absent inflammation and granulation tissue with accentuated collagen formation. Black arrow, neovascularization; blue arrow, collagen formation; yellow arrow, inflammatory cells; green arrow, myofibroblast. Complete re-epithelialization in b, c d & e except a (orange arrow) with dermal damage in (a) the positive control group only (brown arrow).

Figures 3a–e and 4a– e show the re-epithelialization of rat wound healing against *P. aeruginosa* and *K. pneumoniae*, respectively, on day 21. The re-epithelialization was complete with some differences. The animals in all treated groups reformed epidermis except the AgNPs-dHAM dressing group of *P. aeruginosa* and positive control group of *K. pneumoniae*. However, the negative control dressing of treated animals against *K. pneumoniae* displayed the highest thickness of epithelium in comparison to other dressing groups.

Figures 3A–E and 4A–E show the histological features of rat wound healing against *P. aeruginosa* and *K. pneumoniae*, respectively, on day 21 after inducing thermal burns. The animals in the combined colistin + AgNPs-dHAM dressing groups against *P. aeruginosa* and *K. pneumoniae* reported higher collagen scores (3 ± 0; and 3 ± 1, respectively), with mild to absent inflammatory (2 ± 1; and 0 ± 1, respectively) and granulation tissue (2 ± 1; and 0 ± 1, respectively) responses on day 21. The AgNPs-dHAM of both bacteria and colistin-dHAM of *K. pneumoniae* revealed a strong presence of fibroblasts, myofibroblasts and new vessels on day 21, as shown in Table 5.

**Table 5:** Histological features of rat wound healing against *P. aeruginosa* and *K. pneumoniae* on day 21. 0 = Absent, +  = 1, +  +  = 2, +  +  +  = 3.

Groups	Polymorphonuclear neutrophils	Granulation tissue (Fibroblast, Myofibroblast, Neovascularization)	Fibrosis (Collagen fibers)
***P. aeruginosa* (Median ± Range)**			
Positive Control	1 ± 1	2 ± 1	1 ± 1
Negative Control	3 ± 0	2 ± 1	2 ± 1
Colistin-dHAM	1 ± 1	2 ± 1	1 ± 0
AgNPs-dHAM	1 ± 2	3 ± 0	2 ± 1
Colistin + AgNPs-dHAM	2 ± 1	2 ± 1	3 ± 0
***K. pneumoniae* (Median ± Range)**			
Positive Control	2 ± 3	1 ± 1	2 ± 0
Negative Control	2 ± 2	1 ± 1	2 ± 1
Colistin-dHAM	2 ± 2	3 ± 0	1 ± 2
AgNPs-dHAM	1 ± 1	3 ± 1	1 ± 1
Colistin + AgNPs-dHAM	0 ± 1	0 ± 1	3 ± 1

### Wound swabbing results on day 21 against *P. aeruginosa* and *K. pneumoniae*

**Figure 5:**
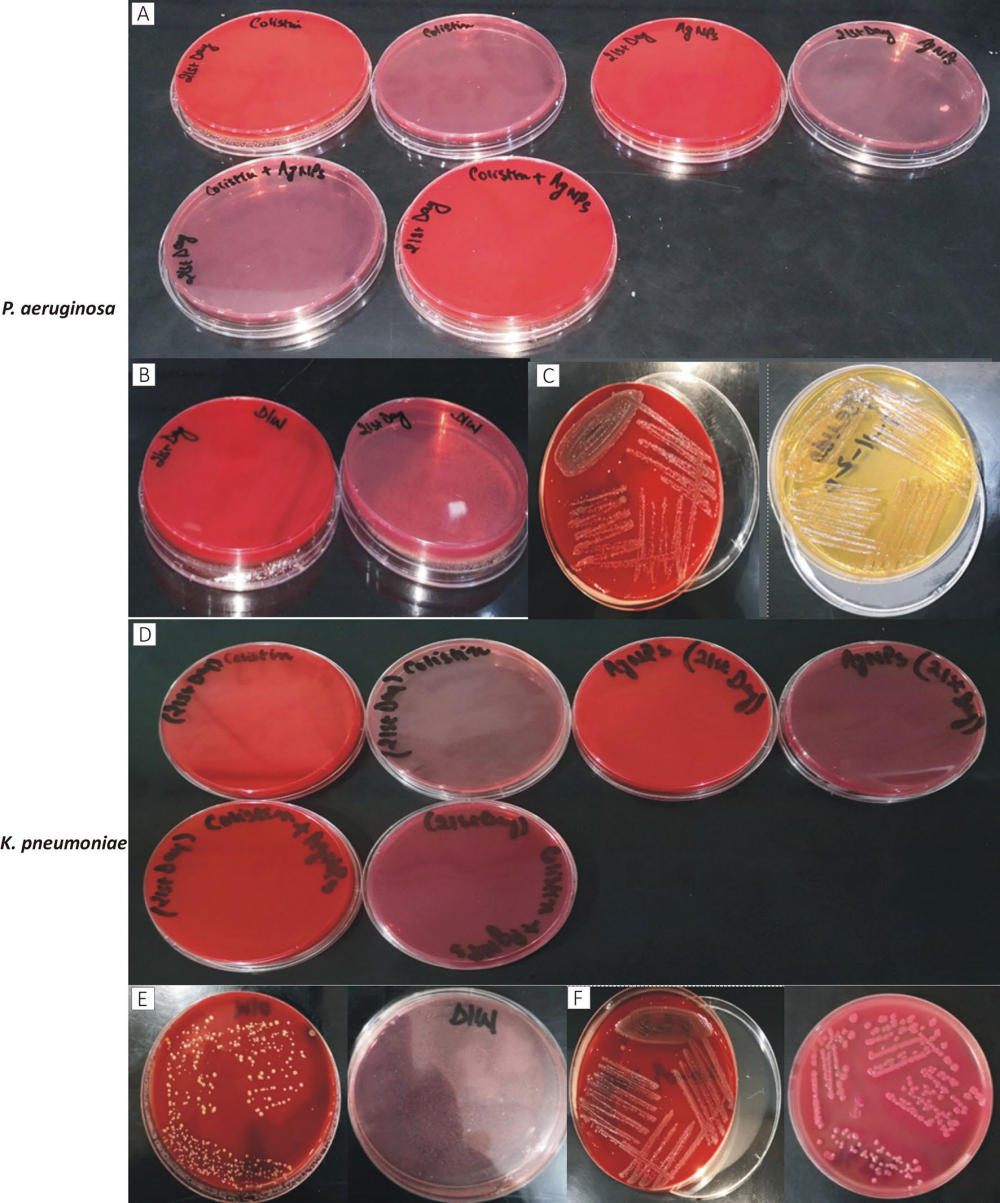
Wound culture of rat wounds on the 21st day. (**A**–**D**) Treated groups: no growth of bacteria in *P. aeruginosa* and *K. pneumoniae* (respectively) cultures, B) no growth of bacteria in negative control of *P. aeruginosa*, (**E**) growth of *S. aureus* in negative control of *K. pneumoniae*, C & F) growth of *P. aeruginosa* and *K. pneumoniae,* respectively in their positive controls of culture media.

Culture swabs from bacterial wounds on the 21st day displayed variable results in negative controls of *P. aeruginosa* and *K. pneumoniae* (Fig. 5). Growth of *S. aureus* was obtained in the negative control of *K. pneumoniae* (Fig. 5E). However, no growth was observed in the negative control of *P. aeruginosa* (Fig. 5B). Furthermore, there was growth of *P. aeruginosa* and *K. pneumoniae* in their respective positive controls, as shown in Fig. 5C and F, respectively. In contrast, rest of the dressing-treated wounds remained negative for bacterial growth.

## Discussion

The present study was aimed to determine the effects of newly designed wound dressings by assessing wound contraction, histopathology and the presence of bacteria.

The results showed that combination treatment with colistin and the AgNPs-dHAM dressing considerably reduced the wound area effectively for *P. aeruginosa* and *K. pneumoniae*. Surprisingly, in the case of *K. pneumoniae*, colistin combined with the AgNPs-dHAM dressing groups showed better wound contraction results than *P. aeruginosa*. The literature proves that using nanoparticles with a smaller diameter (< 30 nm) increases the antibacterial activity of AgNPs against *K. pneumoniae* and *S. aureus*^27^. Marsit et al., (2019) reported excellent antibacterial activity of dried amniotic membranes loaded with polymyxin B against *P. aeruginosa*^20^. There could be several possible explanations for our results. hAM is an appropriate biological scaffold for the seeding and multiplication of cells with an improved capacity to take up antibiotics. Lyophilization has additional, improved adhesion properties^12,28^. Similarly, AgNPs show remarkable antibacterial synergistic effects when combined with colistin and other antibiotics^29,30^. Moreover, AgNPs can easily control bacterial drug resistance. For this purpose, AgNPs employ many bactericidal processes working simultaneously, which could explain the reason for the uncommon resistance of bacteria to silver^31^.

Rapid re-epithelialization is a symbol of healthy wound healing^32^. Regarding histopathological analysis on day 21, complete re-epithelialization and dermal findings were encouraging in the case of *K. pneumoniae*. However, contradictory findings were observed in the case of *P. aeruginosa,* where unexpectedly, the AgNPs-dHAM dressing group failed to re-epithelilalize completely. Similarly, mild to moderate dermal damage was evident in all dressing groups used to treat *P. aeruginosa*-infected burn wounds*.* These findings were almost consistent with a previous study where moderate epidermal necrosis was observed on the 28th day with a moderately disordered arrangement of the dermis ^11^.

Further histopathological analysis of these tissues revealed that the strongest immune cell response was recorded in the negative control group of *P. aeruginosa*. However, the fibrosis was maximum in the combined colistin with AgNPs-dHAM dressing group on day 21 for both bacteria. Complete re-epithelialization with moderate angiogenesis evidence and fibrous tissue was observed in the dermis of AgNP-treated mice on the 28th day^10,33^. Sedighi et al.demonstrated a decrease in inflammatory cells and re-epithelialization after 21 days in the amniotic membrane dressing group in third-degree burn wounds in mice^34^.

Combining colistin with the AgNPs-dHAM dressing accelerated wound healing, probably by decreasing bacterial colonization, as evidenced by the absence of bacteria on day 21 of wound healing. Wound healing is a multistage process that includes three phases. The inflammatory phase is critical for cell debris removal, cytokine release and a comprehensive counter to pathogenic infection. Decreased bacterial counts in tissues minimize the inflammatory phase. The starting point for granulation tissue production and a speedy wound healing is dependent on shortening of the inflammatory step at an early beginning of the proliferative phase^35^. Earlier observations also confirmed the role of AgNPs when combined with other agents, can decrease the duration of wound healing phases^36^.

## Conclusion

Our study concluded pronounced synergistic effects of combined colistin and AgNP-loaded dHAM dressing compared to positive control, negative control, colistin-dHAM and AgNPs-dHAM dressings in rats infected with *P. aeruginosa* and *K. pneumoniae.* This was confirmed by the achievement of faster wound reduction, the presence of considerable fibrosis, complete epithelial reorganization and the absence of bacteria on day 21. The friendly biological synthesis of AgNPs with easy procurement and simple processing of membranes makes them a cost-effective option to be used as a burn dressing commercially due to their improved antibacterial and healing properties. These findings may provide a gateway for other researchers to counteract MDR pathogens in burn wounds and aid clinicians in effectively managing such patients. Immunohistochemistry and molecular aspects were not addressed in our study and this work is not applicable to other species of rats. Further long term in-vitro and in-vivo studies should be carried out to confirm the functionality of the designed wound dressing.

## Supplementary Information

Supplementary Information.

## Data Availability

The data of the current study will be available upon valid request from the authors.

## References

[1] Chen YY (2020). Trends in microbial profile of burn patients following an event of dust explosion at a tertiary medical center. BMC Infect. Dis..

[2] Khezri K, Farahpour MR, Mounesi Rad S (2020). Efficacy of Mentha pulegium essential oil encapsulated into nanostructured lipid carriers as an in vitro antibacterial and infected wound healing agent. Colloids Surf. A Physicochem. Eng. Asp..

[3] El Hamzaoui N, Barguigua A, Larouz S, Maouloua M (2020). Epidemiology of burn wound bacterial infections at a Meknes hospital, Morocco. New Microbes New Infect..

[4] El-Sayed Ahmed MAE-G (2020). Colistin and its role in the Era of antibiotic resistance: An extended review (2000–2019). Emerg Microbes Infect..

[5] Andrade FF, Silva D, Rodrigues A, Pina-Vaz C (2020). Colistin update on its mechanism of action and resistance, present and future challenges. Microorganisms.

[6] Gogry FA, Siddiqui MT, Sultan I, Haq QMR (2021). Current update on intrinsic and acquired colistin resistance mechanisms in bacteria. Front. Med..

[7] Feng X (2021). Synergistic activity of colistin combined with auranofin against colistin-resistant gram-negative bacteria. Front. Microbiol..

[8] Khalil MA (2021). Enhanced efficacy of some antibiotics in presence of silver nanoparticles against multidrug resistant pseudomonas aeruginosa recovered from burn wound infections. Front. Microbiol..

[9] Bruna T, Maldonado-Bravo F, Jara P, Caro N (2021). Silver nanoparticles and their antibacterial applications. Int. J. Mol. Sci..

[10] Paladini F, Pollini M (2019). Antimicrobial silver nanoparticles for wound healing application: Progress and future trends. Materials..

[11] Wasef LG (2020). Effects of silver nanoparticles on burn wound healing in a mouse model. Biol. Trace Elem. Res..

[12] Fénelon M (2021). Applications of human amniotic membrane for tissue engineering. Membranes Basel.

[13] Nouri M (2018). Healing effects of dried and acellular human amniotic membrane and mepitelas for coverage of skin graft donor areas; a randomized clinical trial. Bull. Emerg. Trauma..

[14] Leal-Marin S (2021). Human amniotic membrane: A review on tissue engineering, application, and storage. J. Biomed. Mater. Res..

[15] Naqvi SZH, Zainab SH, Hameed A, Ahmed S, Ali N (2014). Mycogenesis of silver nanoparticles by different Aspergillus species. Sci. Iran..

[16] Cheesbrough M (2006). Microbiological tests.

[17] CLSI. Performance Standards for Antimicrobial Susceptibility and Testing. 30th ed. *CLSI Supplement M100.* Clinical and Laboratory Standards Institute (2020).

[18] Wolfensberger A, Kuster SP, Marchesi M, Zbinden R, Hombach M (2019). The effect of varying multidrug-resistence (MDR) definitions on rates of MDR gram-negative rods. Antimicrob. Resist. Infect. Control..

[19] Naqvi SZ (2013). Combined efficacy of biologically synthesized silver nanoparticles and different antibiotics against multidrug-resistant bacteria. Int. J. Nanomedicine.

[20] Marsit NM (2019). Validation and assessment of an antibiotic-based, aseptic decontamination manufacturing protocol for therapeutic, vacuum-dried human amniotic membrane. Sci. Rep..

[21] Campelo MBD (2018). Effects of the application of the amniotic membrane in the healing process of skin wounds in rats. Acta Cir. Bras..

[22] Ramesh B, Chandrasekaran J, Jeevankumar S, Jacob G, Cherian K (2017). Hybrid amniotic membrane dressing with green silver nanoparticles as bioengineered skin for wounds and burns: A pilot studies. J. Biotechnol. Biomater..

[23] Mencucci R, Menchini U, Dei R (2006). Antimicrobial activity of antibiotic-treated amniotic membrane: An in vitro study. Cornea.

[24] Cardoso AL (2016). Adipose tissue stromal vascular fraction in the treatment of full thickness burns in rats. Acta Cir. Bras..

[25] Rahman MS (2019). Characterization of burn wound healing gel prepared from human amniotic membrane and Aloe vera extract. BMC Complement. Altern. Med..

[26] Tavares Pereira, D. d. S., Lima-Ribeiro, M. H. M., de Pontes-Filho, N. T., Carneiro-Leão, A. M. d. A. & Correia, M. T. d. S. Development of Animal Model for Studying Deep Second-Degree Thermal Burns. *J. Biomed. Biotechnol.* **2012**, 460841 (2012). 10.1155/2012/460841. 10.1155/2012/460841 PMC3379528 22736951

[27] Dakal TC, Kumar A, Majumdar RS, Yadav V (2016). Mechanistic basis of antimicrobial actions of silver nanoparticles. Front. Microbiol..

[28] McDaniel J, Johnson A, Zamora DO (2018). Antibiotic loading of scco2 sterilized and lyophilized human amniotic membrane. Invest. Ophthalmol. Vis. Sci..

[29] Khaled JM (2021). A synergic action of colistin, imipenem, and silver nanoparticles against pandrug-resistant Acinetobacter baumannii isolated from patients. J. Infect. Public Health..

[30] Vazquez-Muñoz R (2019). Enhancement of antibiotics antimicrobial activity due to the silver nanoparticles impact on the cell membrane. PLoS ONE.

[31] Baptista PV (2018). Nano-strategies to fight multidrug resistant bacteria-"A Battle of the Titans". Front. Microbiol..

[32] Farahpour M, Mirzakhani N, Doostmohammadi J, Ebrahimzadeh M (2015). Hydroethanolic Pistacia atlantica hulls extract improved wound healing process; evidence for mast cells infiltration, angiogenesis and RNA stability. Int. J. Surg..

[33] Rani GN, Rao A, Shamili M, JyothiPadmaja I (2018). Combined effect of silver nanoparticles and honey in experimental wound healing process in rats. Biomed. Res..

[34] Sedighi A, Mehrabani D, Shirazi R (2016). Histopathological evaluation of the healing effects of human amniotic membrane transplantation in third-degree burn wound injuries. Comp. Clin. Path..

[35] Ghodrati M, Farahpour MR, Hamishehkar H (2019). Encapsulation of Peppermint essential oil in nanostructured lipid carriers: In-vitro antibacterial activity and accelerative effect on infected wound healing. Colloids Surf. A Physicochem. Eng. Asp..

[36] Oryan A, Alemzadeh E, Tashkhourian J, Nami-Ana SF (2018). Topical delivery of chitosan-capped silver nanoparticles speeds up healing in burn wounds: A preclinical study. Carbohydr. Polym..

