# Author Correction: Synergistic efficacy of colistin and silver nanoparticles impregnated human amniotic membrane in a burn wound infected rat model

**DOI:** 10.1038/s41598-022-20203-w

**Published:** 2022-09-13

**Authors:** Nadia Wali, Aroosh Shabbir, Nadia Wajid, Nasir Abbas, Syed Zeeshan Haider Naqvi

**Affiliations:** 1 Institute of Molecular Biology and Biotechnology (IMBB), The University of Lahore, Defense Road Campus, Lahore, Pakistan; 2 Department of Pathology, Akhtar Saeed Medical and Dental College, Lahore, Pakistan; 3 Department of Statistics, Goverment Graduate College, Jhang, Pakistan

Correction to: *Scientific Reports* https://doi.org/10.1038/s41598-022-10314-9, published online 19 April 2022

The original version of this Article contained errors in Tables 2 and 3. Under the heading ‘Resistant’, the values in the column ‘%age’ were a duplicate of the values in the column ‘proportion’. The correct and incorrect values appear below.

Incorrect Table 2:

**Table:** 

Antimicrobials	Resistant
	%age
Colistin	0.00
Meropenem	0.50
Gentamicin	0.54
Amikacin	0.55
Cefoperazone-sulbactam	0.57
Imipenem	0.53
Piperacillin-tazobactam	0.57
Levofloxacin	0.77
Ciprofloxacin	0.72
Ampicillin-sulbactam	0.85
Cefepime	0.71
Amoxicillin-clavulanic acid	0.57
Ceftazidime	0.62

Correct Table 2:

**Table:** 

Antimicrobials	Resistant
	%age
Colistin	0
Meropenem	50
Gentamicin	54
Amikacin	55
Cefoperazone-sulbactam	57
Imipenem	53
Piperacillin-tazobactam	57
Levofloxacin	77
Ciprofloxacin	72
Ampicillin-sulbactam	85
Cefepime	71
Amoxicillin-clavulanic acid	57
Ceftazidime	62

Incorrect Table 3:

**Table:** 

Antimicrobials	Resistant
	%age
Colistin	0.00
Amikacin	0.52
Gentamicin	0.68
Cefoperazone-sulbactam	0.68
Meropenem	0.70
Imipenem	0.72
Piperacillin-tazobactam	0.76
Levofloxacin	0.82
Ciprofloxacin	0.82
Ampicillin-sulbactam	0.85
Cefepime	0.86
Aztreonam	0.92
Amoxicillin-clavulanic acid	0.96

Correct Table 3:

**Table:** 

Antimicrobials	Resistant
	%age
Colistin	0
Amikacin	52
Gentamicin	68
Cefoperazone-sulbactam	68
Meropenem	70
Imipenem	72
Piperacillin-tazobactam	76
Levofloxacin	82
Ciprofloxacin	82
Ampicillin-sulbactam	85
Cefepime	86
Aztreonam	92
Amoxicillin-clavulanic acid	96

Additionally, in Table 4, under ‘*P. aeruginosa’*, ‘Colistin-dHAM (%age)’, the values for ‘^a^Comparison between and within groups (7th day), *p* value = 0.000*’ and ‘Comparison between and within groups (14th day), *p* value = 0.012*’ were interchanged. The correct and incorrect values appear below.

Incorrect Table 4:

**Table:** 

Days	Statistical measures	Treatment groups (zones of inhibition in mm)
		Colistin-dHAM (%age)
***P. aeruginosa***		
^ a^Comparison between and within groups (7th day), *p* value = 0.000*		
7th day	Mean ± SD ^b^(*p* values)	36.0 ± 6.1 (*p*: 0.001*)
Comparison between and within groups (14th day), *p* value = 0.012*		
14th day	Mean ± SD (*p* values)	32.3 ± 11.0 (*p*: 0.014*)

Correct Table 4:

**Table:** 

Days	Statistical measures	Treatment groups (zones of inhibition in mm)
		Colistin-dHAM (%age)
***P. aeruginosa***		
^ a^Comparison between and within groups (7th day), *p* value = 0.000*		
7th day	Mean ± SD ^b^(*p* values)	32.3 ± 11.0 (*p*: 0.014*)
Comparison between and within groups (14th day), *p* value = 0.012*		
14th day	Mean ± SD (*p* values)	36.0 ± 6.1 (*p*: 0.001*)

The original Article has been corrected.

