# Supplementary material for: Synergistic efficacy of colistin and silver nanoparticles impregnated human amniotic membrane in a burn wound infected rat model
Source: Sci Rep. 2022 Apr 19;12:6414. doi: 10.1038/s41598-022-10314-9 (PMC9018780; doi:10.1038/s41598-022-10314-9)
Supplement: Supplementary file 1 — Supplementary Information. [file 41598_2022_10314_MOESM1_ESM.pdf]

SUPPLEMENTARY INFORMATION

Title: Synergistic efficacy of colistin and silver nanoparticles impregnated human amniotic membrane in a burn wound infected rat model

Authors:

Nadia Wali, Aroosh Shabbir, Nadia Wajid, Nasir Abbas, Syed Zeeshan Haider Naqvi.

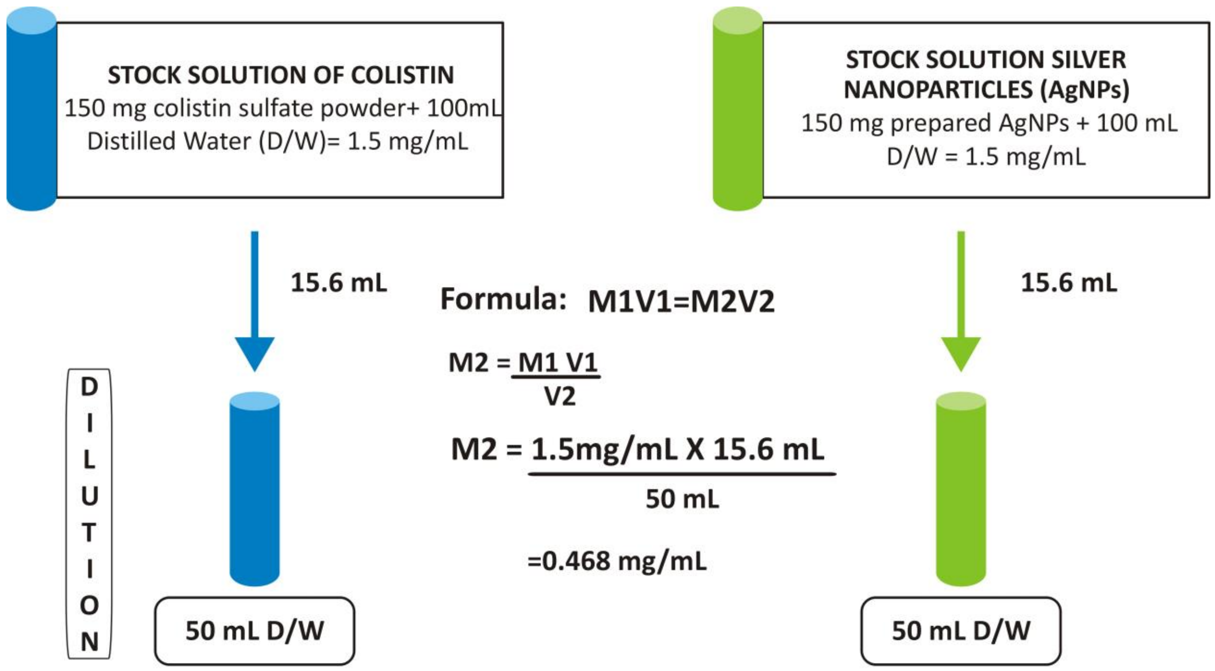

Supplementary Fig. 1. Calculation of colistin & AgNPs dilution
